# Prediction and Prevention of the Mephisto Sign: A Practical Clinical Maneuver for Botulinum Toxin Injections

**DOI:** 10.1111/jocd.71136

**Published:** 2026-08-26

**Authors:** Aslan Yurekli, Ismail Hakki Unal

**Affiliations:** ^1^ Department of Dermatology University of Health Sciences, Gulhane Training and Research Hospital Ankara Turkey

**Keywords:** adverse effects, botulinum toxin, cosmetic techniques, eyebrows, Mephisto sign

To the Editor,

Botulinum toxin is a widely preferred, safe, and effective treatment option in aesthetic dermatology for the correction of forehead, glabellar, and periorbital wrinkles [1]. However, several complications may occur after injection, leading to patient dissatisfaction from an aesthetic perspective. One such complication is the Mephisto sign, which is characterized by a conspicuous elevation of the lateral eyebrows following botulinum toxin injections into the forehead [2]. This phenomenon may result from suppression of the central or medial frontalis muscle by the toxin, leaving the lateral fibers relatively more active [2, 3]. It may occur after central forehead treatment and may also be facilitated by unintended diffusion from glabellar injections into the medial frontalis region. The resulting appearance is frequently interpreted as a “surprised” or “malicious” expression, often prompting corrective injections.

In clinical practice, this complication is usually addressed preventively by applying a low dose of toxin bilaterally to the lateral frontalis region to balance muscle activity [2]. Nevertheless, this approach is not always sufficient for all patients. Human facial musculature varies substantially between individuals: in some, the lateral fibers are markedly dominant, while in others they are less active compared to the medial portion. Differences in forehead height, baseline brow position, frontalis strength, and sex‐related anatomy may further influence treatment outcomes. These variations may be particularly relevant in male patients, in whom lateral frontalis activity may be stronger or extend more superiorly. Such anatomical and functional variations may render standard injection protocols ineffective in certain cases. Therefore, a simple method that enables clinicians to evaluate the balance of frontalis muscle activity prior to injection would be of great importance in optimizing clinical outcomes.

To anticipate the development of the Mephisto sign, we propose a noninvasive clinical maneuver. During examination, the physician gently blocks the patient's medial frontalis with a finger while asking the patient to elevate the eyebrows. A marked rise in the lateral brows under these conditions may indicate a higher tendency toward the Mephisto sign after injection (Figure 1). Conversely, absent or minimal lateral brow elevation during the same maneuver may suggest a lower‐risk pattern (Figure 2). This simple maneuver may serve as a practical adjunct to dynamic pre‐injection assessment and may help the clinician determine whether the lateral fibers should be specifically targeted during the procedure.

**FIGURE 1:**
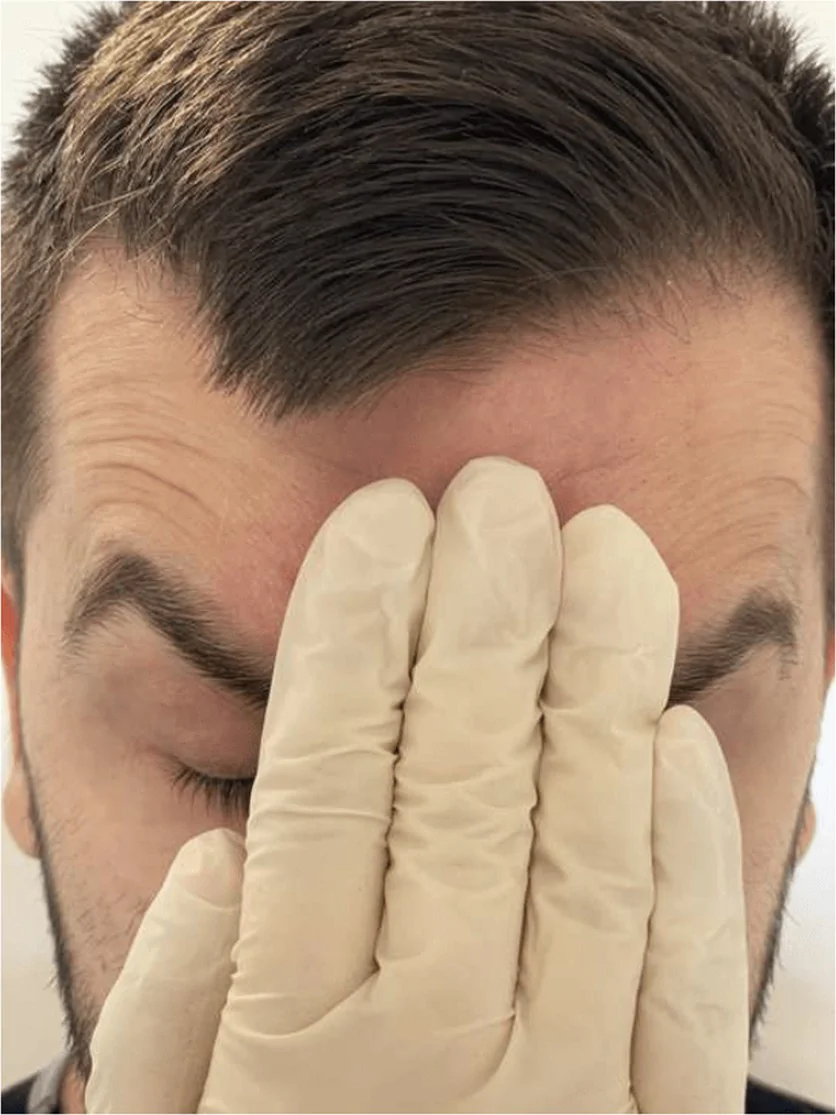
Demonstration of the proposed maneuver in a male patient. Manual blockade of the medial frontalis with fingertip pressure reveals a marked elevation of the lateral brow during attempted eyebrow raising, suggesting a higher tendency toward the development of the Mephisto sign.

**FIGURE 2:**
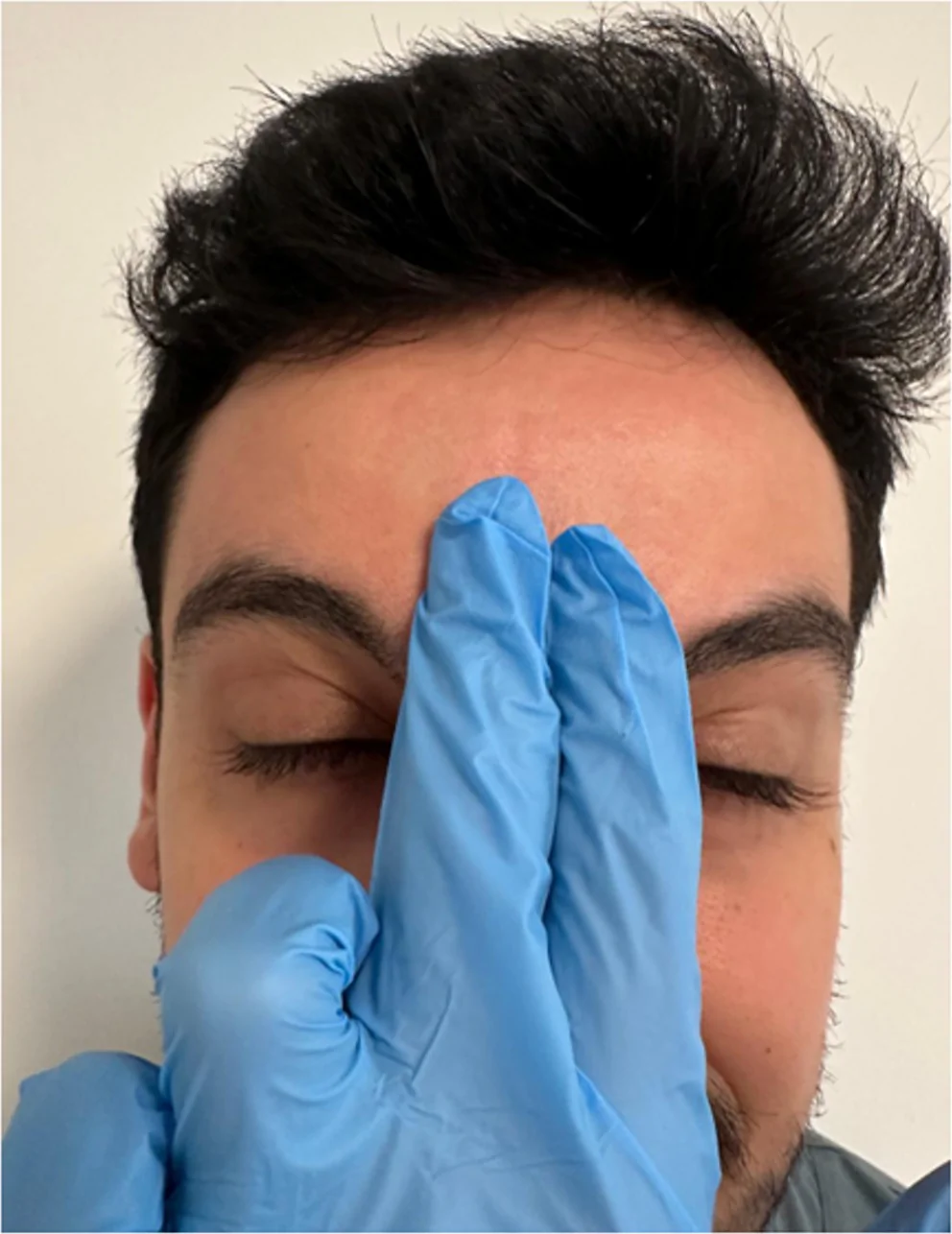
Demonstration of the proposed maneuver in a male patient with less prominent lateral frontalis activity. Manual blockade of the medial frontalis does not produce marked lateral brow elevation, suggesting a lower‐risk pattern for post‐injection lateral brow peaking.

This approach may offer several advantages: it may reduce the risk of post‐injection complications, increase patient satisfaction, and minimize the need for corrective treatments. It is intended to complement, rather than replace, comprehensive pre‐injection evaluation of facial anatomy, dynamic muscle activity, and patient expectations. Furthermore, the maneuver is easy to perform, cost‐free, and can be readily applied in any clinical setting. It may be especially valuable in first‐time botulinum toxin patients, providing preliminary insight into individual muscle dynamics before treatment planning.

In conclusion, success in botulinum toxin applications for aesthetic dermatology depends not only on the technical aspects of injection and dosage but also on tailoring the approach to individual muscle architecture. The proposed pre‐injection maneuver may offer practical predictive value for the occurrence of the Mephisto sign and contributes to a more personalized treatment strategy. Adoption of this method may facilitate more predictable, safe, and aesthetically satisfying outcomes in botulinum toxin procedures.

## Funding

The authors have nothing to report.

## Ethics Statement

This article does not involve animal or interventional human research requiring institutional review board approval.

## Consent

Written informed consent was obtained from the patient for publication of clinical details and accompanying images.

## Conflicts of Interest

The authors declare no conflicts of interest.

## Data Availability

Data sharing is not applicable to this article as no datasets were generated or analyzed during the current study.

## References

[1] H. Sundaram , M. Signorini , S. Liew , et al., “Global Aesthetics Consensus: Botulinum Toxin Type A—Evidence‐Based Review, Emerging Concepts, and Consensus Recommendations for Aesthetic Use, Including Updates on Complications,” Plastic and Reconstructive Surgery 137, no. 3 (2016): 518e–529e, 10.1097/01.prs.0000475758.63709.23. PMC5242214 26910696

[2] E. S. Cho , J. Y. Hwang , and S. T. Kim , “A Proposal to Prevent the ‘Mephisto Sign’ Side Effect of Botulinum Toxin Type A Injection in Chronic Migraine,” Yonsei Medical Journal 54, no. 6 (2013): 1542–1544, 10.3349/ymj.2013.54.6.1542. 24142664 PMC3809864

[3] J. Anido , D. Arenas , C. Arruabarrena , et al., “Tailored Botulinum Toxin Type A Injections in Aesthetic Medicine: Consensus Panel Recommendations for Treating the Forehead Based on Individual Facial Anatomy and Muscle Tone,” Clinical, Cosmetic and Investigational Dermatology 10 (2017): 413–421, 10.2147/CCID.S138274. 29089780 PMC5655032

